# Editorial: 74th annual meeting of the Italian society of physiology: breakthroughs and key discoveries

**DOI:** 10.3389/fphys.2025.1756505

**Published:** 2025-12-05

**Authors:** Myriam Catalano, Roberto Piacentini

**Affiliations:** 1 Department of Physiology and Pharmacology, Faculty of Pharmacy and Medicine, Sapienza University of Rome, Rome, Italy; 2 Department of Neuroscience, Università Cattolica del Sacro Cuore, Rome, Italy; 3 IRCCS Fondazione Policlinico Universitario A. Gemelli, Rome, Italy

**Keywords:** cellular physiology, environmental factors, human physiology, neuro-glial communication, synaptic plasticity

The 74th Annual Congress of the Italian Society of Physiology, which took place in Rome in September 2024, provided a valuable platform for interdisciplinary dialogue, fostering communication among researchers and presenting novel perspectives in physiological research. This Research Topic compiles contributions reflecting the richness of discussions and presentations during the congress, offering an overview of topics of great current interest and scientific relevance.

The articles range from cellular and molecular aspects to neurobiological and systemic processes. They focus on the mechanisms that regulate synaptic plasticity, neuro-glial communication, and the impact of environmental factors on human physiology.

In particular:

Synapse regulation depends on post-translational modifications, including SUMOylation, which modulates the conformation, stability, and localization of synaptic proteins. Bertozzi et al. have demonstrated that sumoylation, a physiological process occurring on proteins, ensures spatiotemporal control of synaptic transmission and plasticity. Conversely, its alteration is associated with the appearance of neurodegenerative illnesses, such as Alzheimer’s diseases, which opens up therapeutic prospects based on SUMO modulation.

Cannata et al. investigated the role of α7 nicotinic acetylcholine receptors (α7-nAChRs) in glutamatergic transmission using a mouse model. Deletion of the gene that encodes these receptors in both neurons and astrocytes, induces a phenotype similar to Alzheimer’s disease (AD), characterized by abnormal β-amyloid accumulation, tau phosphorylation, and neuroinflammation in aged mice (>12 months). However, in young α7-KO mice (4–6 months-old) functional and molecular alterations in glutamatergic transmission, including reduced NMDA currents and altered expression of synaptic proteins, such as Synapsin-1, GluN2A, GluN2B, and the post-synaptic scaffold protein Homer-1 were found. Selective neuronal re-expression of the α7-nAChR gene in α7-KO mice via AAV infection restored post-synaptic functional alterations, but not presynaptic ones. The authors hypothesize that early synaptic dysfunction in Alzheimer’s disease results from an altered cholinergic-glutamatergic dialogue that precedes overt neurodegeneration. They also suggest that clarifying the specific contribution of the α7-nAChR receptor in different cell types could lead to new therapies aimed at preserving circuit functionality in the early stages of Alzheimer’s disease.

Growing global plastic production has made plastic pollution a critical environmental and public health issue, prompting particular attention to nanoplastics (NPs) due to their prevalence and potential biological risks. Giordano et al.'s study investigated the effects of exposure to polyethylene terephthalate (PET) nanoplastics on NIH-3T3 murine fibroblasts, a relevant cell model for processes such as cell migration, which is important for tissue regeneration. The PET NPs realistically represent environmental microplastics because they were obtained using a top-down approach that simulates the mechanical abrasion occurring in the atmosphere. Furthermore, the PET NPs exhibit autofluorescence, which is useful for interaction studies without the use of additional dyes. The results show that PET NPs are internalized by fibroblasts in a dose-dependent manner and localize in the cytoplasm. After 24 h, cell viability decreased by only ≤20% at concentrations of 10–100 μg/mL, but migration was significantly impaired, as evidenced by the scratch assay. This suggests a potential impact on tissue repair. Additionally, exposure induced a dose-dependent increase in reactive oxygen species (ROS), suggesting that intracellular oxidative stress may be a mechanism underlying the migratory deficit. These data underscore the risks that PET NPs pose to cellular functions and highlight the need for further research on the underlying mechanisms.

Loi et al. examined cortical plasticity in older adults using paired associative stimulation (PAS) and new indices were introduced to evaluate the response to stimulation. Curve concavity (CC) was found to be the most effective discriminator between responders and non-responders, significantly correlating with cognitive status as measured by the Montreal Cognitive Assessment (MoCA). The study suggests that CC could be a useful biomarker for monitoring PAS-induced cortical plasticity and its relationship with cognitive status, potentially aiding in the treatment of cognitive disorders.

Among environmental factors modulating endogenous neurogenesis, enriched environment (EE) plays a key role. Marrocco et al. showed that transplanting microbiota from mice raised in an EE to those in a standard environment (SE) decreases anxiety and increases neurogenesis and neurotrophins release in the hippocampus. These findings confirm the role of gut microbiota in mediating the beneficial effects of lifestyle on the brain and suggest prospects for therapeutic interventions based on fecal transplantation for mood disorders.

Masoli et al.’s work is a systematic review of the scientific literature on Purkinje cells, the most complex neurons in the nervous system. These neurons have a vast dendritic tree rich in spines that receive excitatory inputs from parallel and climbing fibers. The study highlighted that, although the total number of spines is lower than initial estimates, they are more efficient given the identification of more complex structures, such as double-headed spines, and functional mechanisms modulated by post-learning morpho-functional changes in glial cells. These recent structural and functional findings on Purkinje cells contribute to synaptic recognition and cerebellar plasticity.

The study by Morrone et al. investigates cross-education, which is the increase in strength of the untrained limb after training the contralateral limb. This process is mediated by interhemispheric dynamics through the corpus callosum. To clarify these mechanisms, the authors employed a lesion model in which nine patients with relapsing-remitting multiple sclerosis and focal lesions of the corpus callosum participated in a six-week, high-intensity, isokinetic training program targeting the dorsiflexors of the stronger ankle. After training, both limbs showed significant increases in strength and reduced asymmetry. However, patients with lesions in the rostral corpus callosum reported less cross-education and reduced improvement in asymmetry between the two limbs. No correlations were found for the other subregions of the corpus callosum. This study underscores the pivotal role of the rostral corpus callosum in mediating cross-education mechanisms and suggests that routinely assessing callosal lesions could inform targeted rehabilitation interventions.

In the review by Porcari et al., glial cell-derived neurotrophic factor (GDNF) emerges as an essential trophic factor for neuronal survival and synaptic plasticity. In addition to protecting dopaminergic neurons, GDNF influences GABAergic hippocampal circuits. It interacts with glial cells and contributes to the regulation of neuroinflammation and the blood-brain barrier. GDNF also plays a role in the peripheral nervous system, controlling the development of sympathetic and parasympathetic fibers, maintaining somatic sensory neurons, and innervating motor neurons at the neuromuscular junction. The importance of GDNF in the biology of many tumors (e.g., neuroendocrine, prostate, and colorectal) has also been emphasized, particularly the fact that the GDNF-RET axis is a double-edged sword that requires precise regulation to achieve therapeutic benefits. GDNF was then explored in depth as a promising therapeutic target for neurodegenerative diseases and other neurological conditions.

Ranieri et al. used the hindlimb unloading (HU) mouse model to evaluate the effects of microgravity on body fluid distribution and kidney function. The kidney is the primary regulator of water and salt balance. Specifically, they analyzed vasopressin levels and renal aquaporin (AQP2) expression. The study showed that 1 week of suspension leads to early activation of the system. After 4 weeks, however, there is inhibition of the process associated with protein degradation mechanisms and reduced translation of AQP2 mRNA. These adaptations reflect physiological strategies that balance central venous pressure and water homeostasis in microgravity conditions. They also provide insight into managing nephropathies associated with prolonged immobility.

Storniolo et al. explore how muscle fatigue affects anticipatory postural adjustments (APAs) when initiating walking. Walking is a task that requires feedforward control of posture coordinated with the focal movement. Sixteen trained men performed walking onset tests before and after a one-minute sequence of counter-movement jumps. Muscle activity was recorded via electromyography, and centre of pressure (CoP) shifts and kinematics were recorded. Fatigue delayed both excitatory and inhibitory APAs, as well as reducing the speed and amplitude of posterior CoP shifts. While most participants maintained a “diving” strategy (bilateral dorsal inhibition and ventral excitation), three subjects adopted a “turning” strategy characterized by reciprocal activations compatible with trunk rotation. These results suggest that the central nervous system possesses a variety of postural strategies that can be selected based on factors such as fatigue, training level, and task constraints.

As Physiology research continues to evolve, it is important to recognize the contributions of scientists and clinicians who drive its progress. The 74th Annual Congress of the Italian Society of Physiology exemplified collaboration and the exchange of ideas that promote the advancement of the biomedical sciences.

We thank all the authors, reviewers, and contributors who made this Research Topic possible.

This Research Topic reflects the curiosity and commitment of the scientific community. We hope the presented results will stimulate new research and bring us closer to understanding the complex mechanisms that regulate human physiology.

